# Genetic Characteristics of Mitochondrial DNA Was Associated with Colorectal Carcinogenesis and Its Prognosis

**DOI:** 10.1371/journal.pone.0118612

**Published:** 2015-03-03

**Authors:** Jae-Ho Lee, Ilseon Hwang, Yu-Na Kang, In-Jang Choi, Dae-Kwang Kim

**Affiliations:** 1 Department of Anatomy, Keimyung University School of Medicine, Daegu, Republic of Korea; 2 Department of Pathology, Keimyung University School of Medicine, Daegu, Republic of Korea; 3 Department of Medical Genetics, Keimyung University School of Medicine, Daegu, Republic of Korea; 4 Hanvit Institute for Medical Genetics, City Women’s Clinic, Buk-gu, Daegu, Republic of Korea; Queen Mary Hospital, HONG KONG

## Abstract

Clinical value of mitochondrial DNA has been described in colorectal cancer (CRC). To clarify its role in colorectal carcinogenesis, mitochondrial microsatellite instability (mtMSI) and other markers were investigated in CRCs and their precancerous lesions, as a multitier genetic study. DNA was isolated from paired normal and tumoral tissues in 78 tubular adenomas (TAs), 34 serrated polyps (SPs), and 100 CRCs. mtMSI, nucleus microsatellite instability (nMSI), KRAS mutation, and BRAF mutation were investigated in these tumors and their statistical analysis was performed. mtMSI was found in 30% of CRCs and 21.4% of precancerous lesions. Mitochondrial copy number was higher in SPs than TAs and it was associated with mtMSI in low grade TAs. KRAS and BRAF mutations were mutually exclusive in TAs and SPs. CRCs with mtMSI showed shorter overall survival times than the patients without mtMSI. In CRCs without nMSI or BRAF mutation, mtMSI was a more accurate marker for predicting prognosis. The genetic change of mitochondrial DNA is an early and independent event in colorectal precancerous lesions and mtMSI and mitochondrial contents are associated with the tubular adenoma-carcinoma sequence, resulting in poor prognosis. This result suggested that the genetic change in mitochondrial DNA appears to be a possible prognosis marker in CRC.

## Introduction

Colorectal cancer (CRC) is common throughout the world, and two pathways of its carcinogenesis have been identified [1–5]. The first pathways involves chromosomal instability (CIN) characterized by sequential accumulation of genetic alterations, such as *APC*, *KRAS*, and *p53* mutations [6–11]. The second pathways entails microsatellite instability (MSI or nMSI) characterized by longer survival, a strong association with *BRAF* mutation, and an inverse correlation with *KRAS* mutation [12–20].

For many years, serrated polyps (SP) have been suggested to exhibit no malignant potential [21]. However, recent proposals have suggested that such lesions with nMSI may be precursors of CRC and may progress through a serrated neoplastic pathway characterized by frequent *BRAF* mutation [22–26]. Due to a lack of studies on these legions, details on the molecular mechanism of such progression remain unclear.

Mitochondrial DNA (mtDNA) differs from nuclear DNA, and multiple copies of mtDNA are present in each mitochondrion. The mutation rate of mtDNA is higher than that of nuclear DNA due to an abundance of reactive oxygen species in the mitochondrial inner membrane, fewer repair mechanisms, and a lack of mtDNA-coating proteins, like histones in the nucleus [27,28]. Previous studies reported a high frequency of mitochondrial microsatellite instability (mtMSI) in various cancers; moreover, mitochondrial and nucleus MSI showed no significant associations [29–31]. In recent studies, researchers have set out to investigate mitochondrial copy number (mtCN) in various cancers [32–35]. These studies showed that mtMSI and mtCN in CRCs have been shown to be closely related to various clinical characteristics; however, there was no study to examine both mitochondrial status and other genetic markers via a multitier approach [36–42].

In the present study, mtMSI was evaluated in CRCs with nMSI exhibiting *KRAS* and *BRAF* mutations. Additionally, their clinicopathological characteristics and potential for prognostic markers were also discussed. To contribute to a better understanding of mitochondrial status during colorectal carcinogenesis, these markers and mtCN were also studied in precursor lesions. This study may help in establishing better treatment of CRCs by indentifying the role of mitochondrial DNA in colorectal carcinogenesis.

## Materials and Methods

### Patients and DNA Extraction

All patients who underwent surgical resection for CRCs at Dongsan Medical Center between 1999 and 2003 were initially considered for enrolment in this study. The exclusion criteria included preoperative chemoradiotherapy, previous history of surgical resection for CRCs, death within 30 postoperative days, and evidence of hereditary non-polyposis colorectal cancer (Amsterdam criteria) or familial adenomatous polyposis. After excluding patients according to these criteria, the Keimyung Human Bio-Resource Bank at Dongsan Medical Center provided 100 paired normal and tumor samples.

To obtain data on the precancerous lesions, the medical records of colonoscopic polypectomies performed between 1999 and 2003 were reviewed retrospectively. All of the pathologic specimens were reviewed by two gastrointestinal pathologists (Hwang and Kang) blinded to knowledge of the clinical data or the results of the molecular assays. The precancerous legions are diagnosed by their microscopic appearance histomorphologically, as a result, it was consisted of 78 TAs and 34 SPs. The study was approved by the Institutional Regional Review Board at Dongsan Medical Center (IRB No.10–157), and informed consent was obtained from all individuals involved in the study. IRB center obtained written consent for the approval.

Tumor areas and adjacent normal mucosa were selected by pathologists from slides of hematoxylin and eosin stained sections. Subsequently, the selected areas from paraffin embedded tissues were used for DNA extraction using a DNA extraction Kit (Absolute DNA extraction Kit, BioSewoom, Korea).

### Mitochondrial and nucleus microsatellite instability

MtMSI was analyzed using eight microsatellite markers ((C)n in D-loop, (CA)n in D-loop, (C)6 in ND1, (A)7 in ND2, (A)7 in COI, (T)7 in COIII, (C)6(A)8 in ND5, and (C)3(A)3 in ND5) as previously described [36,43,44]. Recent studies have demonstrated that BAT25 and BAT26 analysis can accurately detect nMSI without the need for additional markers [45,46], therefore, nMSI was analyzed with two microsatellite markers, BAT25 and BAT 26. Polymerase chain reaction (PCR) was performed and PCR products were denatured in formamide loading buffer and electrophoresed through 7.5% and 10% polyacrylamide gels. Silver stain was performed to develop bands. MSI was defined as either a band shift or the appearance of a novel band in DNA from precancerous or cancerous lesions compared to the paired normal tissues. All experiments were repeated at least twice to rule out any artifacts. Direct DNA sequencing was performed on those PCR products that showed altered band mobility in the above analysis. Variations in the sequences between tumor and matched normal tissue were performed using the ABI 3730 DNA sequencer (Bionics Inc, Korea).

### KRAS and BRAF Mutations

*KRAS* mutations in codons 12 and 13, as well as *BRAF* V600E mutations, were analyzed by pyrosequencing (PyroMark Q24, Sweden). Primers for amplification and pyrosequencing were designed as previously described [47]. The pyrosequencing reaction was performed on a PyroMark Q24 instrument using Pyro Gold Q24 Reagents (Qiagen, Netherlands). The pyrosequencing primers were used in a final concentration of 0.3 μmol/L. Resulting data were analyzed and quantified with PyroMark Q24 software, version 2.0.6 (Qiagen, Netherlands).

### mtDNA copy number

mtDNA copy number (mtCN) was examined using a real-time PCR assay. For the quantitative determination of mtDNA content relative to nDNA, primers for specific amplification of mtDNA COX1 and nDNA-encoded ß-actin gene were selected according to previous studies [32,42,48]. Real-time PCR was then carried out on an LightCycler 480 II system (Roche Diagnostics, Germany) with a total volume of 20 μl of reaction mixture containing 10 μl of SYBR Green Master MIX (Takara, Japan), 8 pmol of each primer, and DNA. The PCR conditions were 95°C for 1 min, followed by 40 cycles of 95°C for 15 s, and 60°C for 30 s. The threshold cycle number (Ct) values of the ß-actin gene and the mitochondrial COXI gene were determined. The copy number of mtDNA in each tested specimen was then normalized against that of ß-actin gene to calculate the relative mtDNA copy number. Each measurement was repeated in triplicate and five serially diluted control samples were included in each experiment.

### Statistical Analysis

SPSS software for Windows was used to conduct al statistical analyses. Relative mtCN was calculated from quantified data and patient subgroups were split based on the median values of T/N ratio (mtCN in tumors divided by that in normal tissue X 100%). Chi-square, Fischer’ exact tests and Student’s t-test were used to analyze the relationship between variables. Survival curves, estimated with the Kaplan—Meier method (Univariate analysis), were compared by log-rank test. Overall survival (OS) was defined as the time between diagnosis and either death from disease or death from other causes. Disease free survival (DFS) was defined as the time between diagnosis and disease recurrence or development of distant metastasis. All *P*-values < 0.05 were considered statistically significant.

## Result

The precursor lesions of CRC consisted of 78 TAs and 34 SPs. The clinicopathological characteristics of the TAs and SPs are presented in S1 Table. TAs were classified into those of low and high grade (LTA and HTA) according to histological features; SPs were categorized as serrated adenomas or hyperplastic polyps. Among SPs, mixed form and traditional serrated adenomas were excluded. The characteristics of the CRCs included in the present study are presented in S2 Table. The clinicopathological characteristics were analyzed according to CRC stage. In doing so, location, N stage, differentiation, and vascular invasion were shown to be significantly different between early and advanced stage CRCs.

### Molecular genetic analysis in Tubular Adenomas and Serrated Polyps

The mtMSI results for 78 TAs and 34 SPs are presented in Table 1. In colorectal precancerous legions, mtMSI was found in 21.4% (24/112) of all lesions, and this was similar between TAs and SPs, regardless of their classification. mtMSI was not associated with any clinicopathological characteristic in both TAs and SPs. Direct sequencing results showed that mtMSI was found in only (C)n (D303–310) and (CA)n (D514) regions of D-loops. Considering the molecular function of D-loops, mtMSI may be associated with mitochondrial content, as a result of mitochondrial replication. Accordingly, subsequent analysis of mtCN revealed significant associations in these legions (Table 2). Higher mtCN was found in SPs (64.7%, 22/34) than in TAs (34.6%, 27/78), a difference that was statistically significant (p = 0.003). Of note, mtMSI was observed in LTAs with higher mtCN (60.0% versus 28.2%), although this did not reach statistical significance (p = 0.059). nMSI was found in 9.0% (7/78) of TAs and 11.8% (4/34) of SPs, respectively. *BRAF* mutation was found in only SPs (20.6%, 7/34), while a higher frequency of *KRAS* mutation was recorded in TAs (23.1%, 18/78) than in SPs (2.9%, 1/34). *KRAS* and *BRAF* mutations were mutually exclusive in TAs and SPs (p < 0.0001). mtMSI and mtCN were not associated with any other clinicopathological characteristics or markers in both TAs and SPs, except for those mentioned above.

**Table 1:** Mitochondrial microsatellite instability (mtMSI) in colorectal precancerous legions.

	LTAs (%, n)	HTAs (%, n)	SPs (%, n)
Total	20.4 (10/49)	24.1 (7/29)	20.6 (7/34)
Gender			
Male	20.0 (7/35)	27.8 (5/18)	21.7 (5/23)
Female	21.4 (3/14)	18.2 (2/11)	18.2 (2/11)
Location			
Colon	15.4 (2/13)	37.5 (3/8)	37.5 (3/8)
Rectal	22.2 (8/36)	19.0 (4/21)	15.4 (4/26)
mtCN			
Low	12.5 (4/32)	31.6 (6/19)	8.3 (1/12)
High	35.3 (6/17)	10.0 (1/10)	27.3 (6/22)
nMSI			
(-)	20.0 (9/45)	23.1 (6/26)	20.0 (6/30)
(+)	25.0 (1/4)	33.3 (1/3)	25.0 (1/4)
*KRAS* *			
(-)	17.5 (7/40)	20.0 (4/20)	21.2 (7/33)
(+)	33.3 (3/9)	33.3 (3/9)	0 (0/1)
*BRAF* *			
(-)	100 (49/49)	100 (29/29)	18.5 (5/27)
(+)	0 (0/49)	0 (0/29)	28.6 (2/7)

LTA, low grade of tubular adenoma; HTA, high grade of tubular adenoma; SP, serrated polyp.

* LTA and HTA versus SP, p < 0.001

**Table 2:** Mitochondrial copy number (mtCN) in colorectal precancerous legions.

	LTAs (n = 49)		HTAs (n = 29)		SPs (n = 34)	
	Low (n, %)	High (n, %)	Low (n, %)	High (n, %)	Low (n, %)	High (n, %)
Total *	32 (65.3)	17 (34.7)	19 (65.5)	10 (34.5)	12 (35.3)	22 (64.7)
Gender						
Male	22 (62.9)	13 (37.1)	14 (12.7)	4 (19.4)	8 (34.8)	15 (65.2)
Female	10 (71.4)	4 (28.6)	5 (45.5)	6 (54.5)	4 (36.4)	7 (63.6)
Location						
Colon	10 (71.4)	4 (28.6)	5 (62.5)	3 (37.5)	3 (37.5)	5 (62.5)
Rectal	22 (62.9)	13 (37.1)	14 (66.7)	7 (33.3)	9 (34.6)	17 (65.4)
mtMSI						
(-)	28 (71.8)	11 (28.2)	13 (59.1)	9 (40.9)	11 (40.7)	16 (59.3)
(+)	4 (40.0)	6 (60.0)	6 (85.7)	1 (14.3)	1 (14.3)	6 (85.7)
nMSI						
(-)	31 (68.9)	14 (31.8)	18 (64.3)	8 (30.8)	11 (36.7)	19 (63.3)
(+)	1 (25.0)	3 (75.0)	1 (33.3)	2 (66.7)	1 (25.0)	3 (75.0)
*KRAS*						
(-)	28 (70.0)	12 (30.0)	13 (65.0)	7 (35.0)	12 (36.4)	21 (63.6)
(+)	4 (44.4)	5 (55.6)	6 (6637)	3 (33.3)	0 (0)	1 (100)
*BRAF*						
(-)	31 (65.3)	17 (34.7)	19 (65.5)	10 (34.5)	10 (37.0)	17 (63.0)
(+)	-	-	-	-	2 (28.6)	5 (71.4)

LTA, low grade of tubular adenoma; HTA, high grade of tubular adenoma; SP, serrated polyp.

* LTA and HTA versus SP, p = 0.003

### Molecular genetic analysis in Colorectal Cancers

The results of molecular genetic analysis in 100 CRCs are presented in Table 3. mtMSI was found in 30.0% (30/100) of CRCs. Most mtMSI was found in D-loops, and the distribution thereof was similar to that in the precursor lesions. Patients with mtMSI tended to show a lower frequency (15.4%) of *KRAS* mutations than those without mtMSI (35.1%), although this was not statistically significant (p = 0.06). Overall, mtMSI was not associated with any clinicopathological characteristic in CRCs. However, nMSI, *KRAS* and *BRAF* mutations showed clinicopathological characteristics when stratifying for stage (data not shown). Briefly, in early stage CRCs, patients with mtMSI exhibited features of vascular invasion (p = 0.03). Additionally, nMSI was shown to be associated with male sex (p = 0.02) and vascular invasion (p = 0.05). In advanced stage CRCs, rectal cancers showed a higher frequency of KRAS mutation (p = 0.03); meanwhile, BRAF mutation was related to female sex (p = 0.04) and less lymph node invasion (p = 0.05). When stratifying for stage, *KRAS* and *BRAF* mutations held no further clinicopathological significance, except for the variables described above.

**Table 3:** Clinicopathological Characteristics of Colorectal Cancers According to Genetic Status.

	Total (n)	mtMSI (n, %)	nMSI (n, %)	*KRAS* (n, %)	*BRAF* (n, %)
Total	100	30 (30)	16 (16)	26 (26)	7 (7)
Gender			P = 0.022*		
Male	65	18 (29.0)	14 (22.6)	14 (22.6)	2 (3.2)
Female	38	12 (31.6)	2 (5.3)	12 (31.6)	5 (13.2)
Location					
Colon	41	14 (34.1)	5 (12.2)	8 (19.5)	5 (12.2)
Rectal	59	16 (27.1)	11 (18.6)	18 (30.5)	2 (3.4)
Stage					
I,II (early)	44	11 (25.0)	5 (11.4)	8 (18.2)	4 (9.1)
III,IV (advanced)	56	19 (33.9)	11 (19.6)	18 (32.1)	3 (5.4)
T stage					
T1	1	0 (0)	0 (0)	1 (100)	0 (0)
T2	16	6(37.5)	3 (18.7)	2 (12.5)	1 (6.2)
T3	74	20 (27.0)	11 (14.9)	21 (28.4)	6 (8.1)
T4	9	4 (44.4)	2 (22.2)	2 (22.2)	0 (0)
N stage					
N0	47	13 (27.7)	5 (9.4)	8 (17.0)	5 (10.6)
N1	30	11 (36.7)	4 (13.3)	11 (36.7)	2 (6.7)
N2	23	6 (26.1)	7 (30.4)	7 (30.4)	0 (0)
M stage					
M0	88	27 (30.7)	14 (15.7)	23 (25.8)	6 (6.7)
M1	10	2 (20.0)	2 (18.2)	3 (27.3)	1 (9.1)
Differentiation					
Well/Moderate	91	27 (29.7)	13 (14.3)	26 (28.6)	6 (6.6)
Poor/Undifferentiated	9	3 (33.3)	3 (33.3)	0 (0)	1 (11.1)
Vascular invasion					
(+)	66	22 (33.3)	14 (21.2)	19 (30.3)	5 (7.6)
(-)	34	8 (23.5)	2 (5.9)	7 (17.6)	2 (5.9)
mtMSI					
(-)	70	-	14 (20.0)	22 (31.4)	5 (7.1)
(+)	30	-	2 (6.7)	4 (13.3)	2 (6.7)
nMSI					
(-)	84	28 (33.3)	-	20 (23.8)	7 (8.3)
(+)	16	2 (12.5)	-	6 (37.5)	0 (0)
*KRAS*					
(-)	74	26 (35.1)	10 (13.5)	-	4 (5.4)
(+)	26	4 (15.4)	6 (23.1)	-	3 (11.5)
*BRAF*					
(-)	93	28 (30.1)	16 (17.2)	23 (24.7)	-
(+)	7	2 (28.6)	0 (0)	3 (42.9)	-

*p < 0.05

### Survival Analysis in Colorectal Cancers

**Fig 1:**
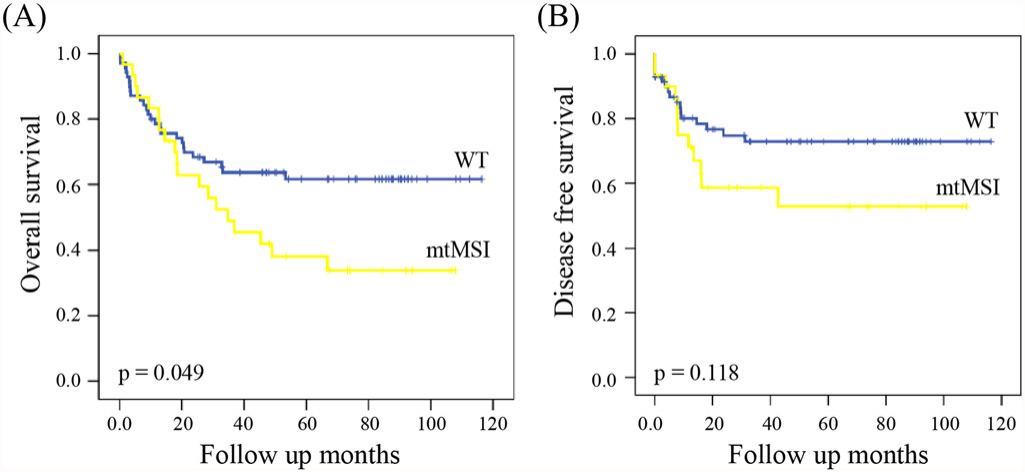
Kaplan—Meier curves for overall survival (A) and disease free survival (B) of colorectal cancer patients according to mitochondrial microsatellite instability status.

The median follow-up of patients for survival analysis was 47.5 months (1–113). The results of the univariate analysis in CRCs are presented in S3 Table. Kaplan-Meier curve revealed mtMSI to be a significant prognostic marker for OS but not DFS (Fig. 1). OS in patients with mtMSI was shorter than in those without mtMSI (HR 3.88; 95% CI, 0.25–0.89; p = 0.049). When stratifying for variables, mtMSI was shown to be a statistically significant prognostic marker for OS (S1 Fig.): mtMSI conferred a poor prognosis in colon cancer (HR 4.86; 95% CI, 0.15–0.87; p = 0.027). In CRCs without nMSI or BRAF mutation, mtMSI was also an accurate marker for predicting OS (HR 4.75; 95% CI, 0.24–0.87; p = 0.029 without nMSI, HR 4.38; 95% CI, 0.23–0.88; p = 0.036 without *BRAF* mutation).

## Discussion

Colorectal cancer is considered a heterogeneous disease, as CRCs and their precursors exhibit distinct pathological features and molecular signatures. To identify colorectal carcinogenesis and potential markers for the prognosis thereof, multitier genetic approaches have been performed to investigate CRCs and their precursors [22–26]. Nevertheless, although mitochondrial microsatellite instability has garnered focus as a cancer marker, no multitier study including mtMSI has been conducted in colorectal tumors [29–37]. Therefore, the present study attempted to investigate the clinicopathological characteristics and prognosis of CRCs with mtMSI for the first time.

In the present study, mtMSI was found in 30% of CRCs, which was similar to that reported in previous studies [37–40]. A recent study of a Korean population showed that mtMSI was found in over half (59%) of the patients [49]. This difference might be caused by different experimental methods and sample selection. Notwithstanding, none of the previous studies, nor the present study, confirmed an association between mtMSI and the clinicopathological characteristics of CRCs. Instead, CRCs with mtMSI in the present study were shown to be associated with shorter overall survival (OS), and this result was in agreement with the results reported by Lievre et al. [38]. The study about the prognosis of CRCs with mtMSI was rare and their results were considered controversial until now. Previously, Chang et al. [41] reported that mtMSI had no significant impact on prognosis. Inversely, Tsai et al. [40] showed a better prognosis in *Dukes* stage C CRCs with mtMSI. Recent research in a Korean population revealed a higher frequency of mtMSI in tumors of larger size and of more advanced TNM stage, suggesting mtMSI as a risk factor for poor outcomes [49]. Additionally, other studies suggested that only nMSI and KRAS and BRAF mutations may be significant markers for prognosis in CRCs [19,20,47]. However, our results were not in agreement with these studies. Instead, we showed that combined analysis of mtMSI, nMSI, and BRAF mutation could accurately predict the prognosis of CRCs. Therefore, we cannot rule out the prognostic value of nMSI and BRAF mutations.

To clarify how mtMSI contributes to colorectal development and prognosis, we investigated genetic markers thereof in colorectal precancerous legions. A similar frequency of mtMSI was found in TAs and SPs, and mtMSI was not shown to be associated with their characteristics or with KRAS or BRAF mutations. Most mutations were found in the D-loop, a noncoding sequence of the mitochondrial genome important in mtDNA replication and transcription [30–34]. Within this region, (C)n repeat (D310 sequence) is an essential element for mtDNA replication because it contains the H-strand replication origin [50]. Accordingly, we investigated mtCN in these legions, as well as the relationship between mtCN and mtMSI. The results revealed a higher mtCN in SPs than TAs; mtCN was not associated with other markers. Interestingly, increased mtCN tended to be associated with mtMSI in LTAs, although this did not reach statistical significance (p = 0.059). These results indicated that mtMSI in LTA may drive higher mitochondrial content, suggesting that mitochondrial DNA status may contribute to the tubular adenoma-carcinoma sequence more than the serrated pathway. In the present study, mtMSI was more significantly associated with the prognosis of CRCs without nMSI or BRAF mutation. This result supports our assertion about its involvement with tubular adenoma-carcinoma sequence, as nMSI and BRAF mutation are representative genetic markers of the serrated pathway.

**Fig 2:**
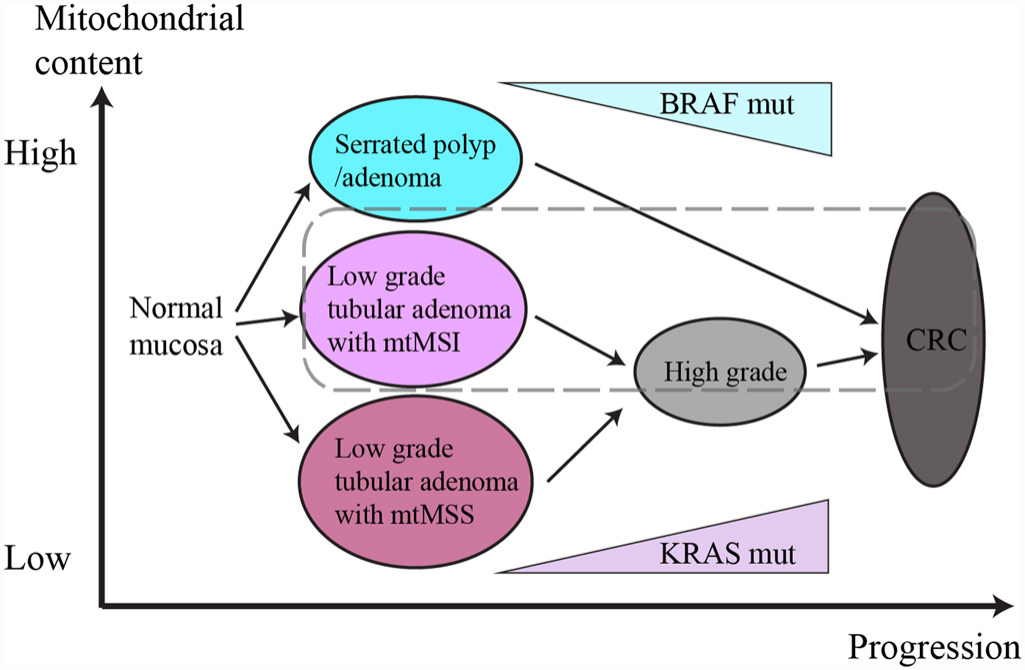
Schematic diagram of the role of mitochondrial DNA in colorectal carcinogenesis. MtMSI is involved in the progression of tubular adenomas as an early event. According to mtMSS and mtMSI, low grade tubular adenomas have different mitochondrial content. And tubular adenomas with mtMSI drive independent carcinogenesis pathway, resulting poor prognosis (dashed line).

Lee et al. [32] suggested that mtMSI was associated with decreased mtCN in hepatocellular carcinoma. However, Guo et al. [51] reported recently that mtCN in specimens with mtMSI was significantly higher than in those without mtMSI in laryngeal squamous cell carcinoma. Our study demonstrated increased mtCN in LTAs with mtMSI, and these data indicated that changes in mtCN as a result of mtMSI may differ from tumor to tumor. Based on these data, we deduced the role of mitochondrial DNA in colorectal carcinogenesis as presented in Fig. 2. Accordingly, changes in mitochondrial DNA status may drive the progression course of colorectal mucosa to TA or SP and may also cause differences in prognosis. These data suggested a new paradigm for colorectal carcinogenesis; nevertheless, further study with a larger number of samples is needed.

In conclusion, this is the first study to suggest the role of mitochondrial DNA in colorectal carcinogenesis to the best of our knowledge. The data herein indicated that mtMSI is an early event in the tubular adenoma-carcinoma sequence, resulting in poor prognosis. In the era of personalized cancer therapy, further study of the characteristics of mitochondrial DNA in precancerous legions may lead to better prognosis and treatment thereof, considering the importance of mitochondria in anticancer drug development.

## Supporting Information

S1 Table Clinicopathological Characteristics of the Patients with Tubular Adenomas and Serrated Polyps. (DOC)

S2 Table Clinicopathological Characteristics of the Patients with Colorectal Cancers According to Stage. (DOCX)

S3 Table Univariate Analysis for Overall Survival and Disease Free Survival of Patients with colorectal Cancer. (DOCX)

S1 Fig Kaplan—Meier curves for overall survival according to mitochondrial microsatellite instability status. (DOC)
