# Supplementary material for: Genetic Characteristics of Mitochondrial DNA Was Associated with Colorectal Carcinogenesis and Its Prognosis
Source: PLoS One. 2015 Mar 3;10(3):e0118612. doi: 10.1371/journal.pone.0118612 (PMC4348484; doi:10.1371/journal.pone.0118612)
Supplement: S1 Table — (DOC) [file pone.0118612.s001.doc]

Table S1. Clinicopathological Characteristics of the Patients with Tubular Adenomas and Serrated Polyps

|  | No. of patients (%) | | p |
| --- | --- | --- | --- |
|  | TA (n = 78) | SP (n = 34) |
| Age (mean) | 59.97 | 56.71 | 0.28 |
| Gender |  |  | 0.97 |
| Male | 53 (67.9) | 23 (67.6) |  |
| Female | 25 (32.1) | 11 (32.4) |  |
| Location |  |  | 0.81 |
| Right | 20 (25.6) | 8 (23.5) |  |
| Left | 58 (74.4) | 26 (76.5) |  |
| Dysplasia |  |  |  |
| Low grade | 49 (62.8) | - |  |
| High grade | 29 (37.2) | - |  |
| Type |  |  |  |
| HP | - | 16 (47.1) |  |
| SA | - | 18 (52.9) |  |

TA: tubular adenoma; SP: serrated polyp; SA: serrated adenoma; HP: hyperplastic polyp.
