# Supplementary material for: Genetic Characteristics of Mitochondrial DNA Was Associated with Colorectal Carcinogenesis and Its Prognosis
Source: PLoS One. 2015 Mar 3;10(3):e0118612. doi: 10.1371/journal.pone.0118612 (PMC4348484; doi:10.1371/journal.pone.0118612)
Supplement: S2 Table — (DOCX) [file pone.0118612.s002.docx]

Table S2. Clinicopathological Characteristics of the Patients with Colorectal Cancers According to Stage

|  | No. of patients (%) | | | p |
| --- | --- | --- | --- | --- |
|  | Total | Early stage | Advanced stage |  |
| Age (years, mean) | 68.01 | 68.84 | 67.12 | 0.47 |
| Gender |  |  |  | 0.33 |
| Male | 62 (62) | 29 (67.4) | 33 (57.9) |  |
| Female | 38 (38) | 14 (32.6) | 24 (42.1) |  |
| Location |  |  |  | <0.0001 |
| Colon | 41 (41) | 27 (62.8) | 14 (24.6) |  |
| Rectal | 59 (59) | 16 (37.2) | 43 (75.4) |  |
| T stage |  |  |  | 0.15 |
| T1 | 1 (1) | 1 (2.3) | - |  |
| T2 | 16 (16) | 7 (16.3) | 9 (15.8) |  |
| T3 | 74 (74) | 34 (79.0) | 40 (70.2) |  |
| T4 | 9 (9) | 1 (2.3) | 8 (14.0) |  |
| N stage |  |  |  | <0.0001 |
| N0 | 46 (46) | 43 (100) | 3 (5.3) |  |
| N1 | 30 (30) | - | 30 (52.6) |  |
| N2 | 24 (24) | - | 24 (42.1) |  |
| Differentiation |  |  |  | 0.042 |
| Well/Moderate | 91 (91) | 42 (97.7) | 49 (86) |  |
| Poor/Undifferentiated | 9 (9) | 1 (2.3) | 8 (14) |  |
| Vascular invasion |  |  |  | <0.0001 |
| (＋) | 66 (66) | 19 (44.2) | 47 (82.5) |  |
| (－) | 34 (34) | 24 (55.8) | 10 (17.5) |  |
