# Supplementary material for: Genetic Characteristics of Mitochondrial DNA Was Associated with Colorectal Carcinogenesis and Its Prognosis
Source: PLoS One. 2015 Mar 3;10(3):e0118612. doi: 10.1371/journal.pone.0118612 (PMC4348484; doi:10.1371/journal.pone.0118612)
Supplement: S3 Table — (DOCX) [file pone.0118612.s003.docx]

Table S3. Univariate Analysis for Overall Survival and Disease Free Survival of Patients with colorectal Cancer

| Variable | n | OS (%) | p | DFS (%) | p |
| --- | --- | --- | --- | --- | --- |
| Gender |  |  | 0.84 |  | 0.52 |
| Male | 62 | 56.5 |  | 72.6 |  |
| Female | 38 | 52.6 |  | 68.4 |  |
| Location |  |  | 0.23 |  | 0.06 |
| Colon | 41 | 61.0 |  | 80.5 |  |
| Rectal | 59 | 50.8 |  | 64.4 |  |
| Stage |  |  | 0.48 |  | <0.001 |
| Early | 44 | 56.8 |  | 90.9 |  |
| Advanced | 56 | 53.6 |  | 55.4 |  |
| T stage |  |  | 0.07 |  | 0.002 |
| T1 | 1 | 100 |  | 100 |  |
| T2 | 16 | 81.2 |  | 93.8 |  |
| T3 | 74 | 51.4 |  | 70.3 |  |
| T4 | 9 | 33.3 |  | 33.3 |  |
| N stage |  |  | 0.012 |  | <0.001 |
| N0 | 47 | 55.3 |  | 87.2 |  |
| N1 | 30 | 70.0 |  | 63.3 |  |
| N2 | 23 | 34.8 |  | 47.8 |  |
| Differentiation |  |  | 0.005 |  | 0.47 |
| Well/Moderate | 91 | 58.2 |  | 71.4 |  |
| Poor/Undifferentiated | 9 | 22.2 |  | 66.7 |  |
| Vascular invasion |  |  | 0.11 |  | 0.005 |
| Present | 66 | 50.0 |  | 62.1 |  |
| Absent | 34 | 64.7 |  | 88.2 |  |
| mtMSI |  |  | 0.049 |  | 0.118 |
| Wild | 70 | 62.9 |  | 75.7 |  |
| Mutant | 30 | 36.7 |  | 60.0 |  |
| nMSI |  |  | 0.92 |  | 0.76 |
| Wild | 84 | 54.8 |  | 70.2 |  |
| Mutant | 16 | 56.2 |  | 75.0 |  |
| *KRAS* |  |  | 0.17 |  | 0.33 |
| Wild | 74 | 51.4 |  | 74.3 |  |
| Mutant | 26 | 65.4 |  | 61.5 |  |
| *BRAF* |  |  | 0.90 |  | 0.12 |
| Wild | 93 | 54.8 |  | 68.8 |  |
| Mutant | 7 | 57.1 |  | 100 |  |

OS, overall survival; DFS, disease free survival.
