# Supplementary material for: Genetic Characteristics of Mitochondrial DNA Was Associated with Colorectal Carcinogenesis and Its Prognosis
Source: PLoS One. 2015 Mar 3;10(3):e0118612. doi: 10.1371/journal.pone.0118612 (PMC4348484; doi:10.1371/journal.pone.0118612)

Fig. S1. Kaplan–Meier curves for overall survival according to mitochondrial microsatellite instability status. (A) In colon cancer patients (B) In nMSS patients (C) In BRAF mutation (－) patients


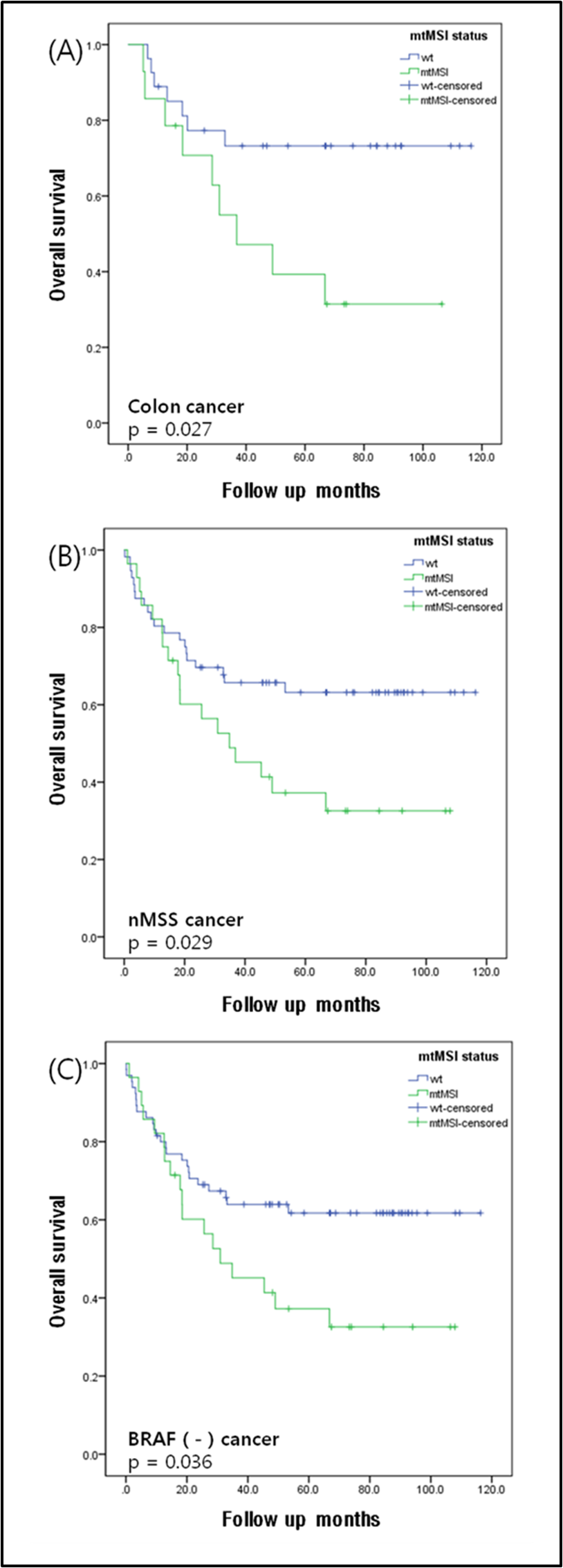

Supplement: S1 Fig — (DOC) [file pone.0118612.s004.doc]
